# Editorial Chit-Chat

**Published:** 1871-01

**Authors:** 


### EDITORIAL CHIT-CHAT.

A WOMAN DOCTOR.—Mrs. Hannah E. Longshore, M. D., of Philadelphia, has a very large and paying practice. Her income is more than $10,000 a year.

QUITE PROPER.—They have ceased singing the irrepressible Marseillaise, in Paris, since the Prusians have surrounded the city. Their favorite song now is "Up in a balloon, boys."

QUEER SQUIRRELS.—The Indianapolis *Journal* says there is an octogenarian in that state, who shoots squirrels without spectacles. There must be very few squirrels wearing spectacles, out there, this season.

THE NORTHERN CENTRAL ROAD.—This excellent road is in fine condition this winter. The trains are making good time, are loaded with passengers, and is the most direct route to Harrisburg, Philadelphia and Baltimore. See time table.

A NEW MUSCLE.—The Demonstrator of Anatomy in Harvard Medical College has discovered a new muscle, hitherto undescribed in any of the works. He is about taking out a patent, and will allow no one to use the muscle without paying royalty.

THAT DROP CURTAIN.—Our beautiful place of amusement—the Opera House—is about to take on the appearance of a fifth rate lager bier garden, by the introduction of a drop curtain covered all over with advertising cards. It's a shame—thought the managers of the Opera House had better taste.

LATER.—That Drop Curtain will not be completed—it is taken out of the *way*.

LAST NUMBER. — This is the last number of Volume VI, of the BISTOURY. Hope our friends will remit promptly, for the next volume. Only fifty cents, remember, and we will agree to give you more than the worth of your money in every number.

LEHIGH VALLEY RAIL ROAD.—The trains are now running over this road from this city to New York and Philadelphia. It is by far the most beautiful and safest route to New York, and reaches the city as soon as do the trains on the Erie. Ask for tickets *via* the "Lehigh Valley Road." Time table will be found in the BISTOURY.

PAMPHLETEERING.—Drs. L. A. Sayre and A. Ruppanuer, of New York, are accusing each other of unprofessional conduct, in a number of pamphlets published against each other. It is difficult to tell who has the best of the fight, but it is amusing for an outsider to look on and enjoy the fun.

SLEEPY.—The order on the Erie forbidding breakmen from riding inside the coaches, was intended to keep them awake, as they were in the habit of sleeping, and not hearing the "toot!" of down breaks.

METALS FOR FUEL.—A scientific Englishman proposes to burn iron, zinc, or manganese instead of coal on our large ocean steamers. He claims that one pound of zinc will evaporate more than four times as much water as one pound of coal, the advantage of which, on a long sea voyage, is obvious.

WARNING TO LADIES.—One of our exchanges says that black silk is liable to spontaneous combustion. We remember of having frequently seen sparks around a package of black silk, but believe as a general thing, there was a woman in it. We have never supposed that there was any chemical mystery about *such* a package, however.

WOODEN NUTMEGS OUTDONE.—The *Manufacturer & Builder* informs its readers that coffee beans are now produced by an artificial process. The material is a pale greenish clay, which is moulded into a form resembling ordinary coffee, by a machine constructed after the fashion of bullet moulds.

This exceedingly interesting product can be manufactured for one cent a pound, so that it is likely that the staple product—burnt peas, or rye, will be completely driven out of the market.

THE BRIXTON BABY FARMER.—The villianous woman by the name of Margaret Waters, who was imprisoned and tried at Brixton, Eng., for causing the death of a large number of illegitimate children, committed to her charge, was righteously hung on the 11th of October. She confessed to having caused their deaths by neglect and the administration of unsuitable food, literally starving the little innocents to death.

Hanging was altogether too easy and comfortable a death for such a fiend.

THE SIAMESE TWINS.—These poor fellows are in a "peck o' trouble." One of them is suffering from an attack of hemiplegia, completely paralysing one half his person, while the other is in perfect health. Should the paralysed one die, as he is greatly in danger of doing, the well one will have to lay down and do likewise, unless some surgeon can safely separate him from his dead brother.

---

WAKE HER UP.—The "Sleeping Beauty" of Tennessee, who has passed twenty-one years in a state of somnolence, with the exception of occasional wakeful moments for purposes of nourishment, is now stopping in St. Louis, and is being "interviewed" by the Physicians of that city. It is thought that even if their treatment produces no beneficial result, that certainly the "rousing bill" which they are likely to present, will certainly make her "open her eyes."

---

NILSSON.—This lady is creating a great furore in New York and Boston. She is introducing an entirely new style of responding to *encores*. The manner in which she sang "Down on the Swaunee River" and "O, Susanna," fairly set the devotees of classical music "mad with delight." She is going to Cincinnati, when we expect to hear that she will respond to the applause of the inhabitants of that celebrated metropolis, with the dulcet notes of the "Ham-Fat Man" or "Root Hog or Die."

---

MISSIONARIES IN THE GERMAN ARMY.—The German Surgeons complain loudly against the ill-timed zeal of the missionaries who visit their hospitals, distributing tracts among the wounded, talking to them of their probable death, and calling on them to repent their sins. The Surgeons object to this proceeding, for the reason that the wounded require to be kept quiet and in good spirits, in order to make a speedy recovery. They have caused orders to be issued forbidding the admission of the missionaries to the sick wards.

---

EXPENSIVE WATER.—Prof. Chandler, chemist to the New York Board of Health, in his report on the milk supply of the city, says that for every three quarts of milk sold in the city of New York, one quart of water is sold as a dilution. He estimates that there is 120,000,000 quarts of milk used, and 40,000,000 quarts of water to dilute it with. Therefore, Croton water, in milk, costs the buyers about $4,000,000 annually, or about $12,000 every blessed day. Croton and Milk is, therefore, almost as expensive as Gin and Milk, and it is not to be wondered at, that their ministers should prefer the latter at the same cost.

---

LIFE ASSURANCE EXAMINATIONS.—Such examinations, as at present conducted, are mere burlesques. No intelligent physician can afford to make a thorough examination of the condition of a man's health for the small sum of three dollars. The fee is too small—the physician cannot afford to spend the time necessary to make such an examination, as would be of the least service to, or answer the purpose intended by Insurance Companies. Physicians should therefore, refuse to conduct examinations for such fees. It is a fraud upon al concerned. The Physician, the company and the public are defrauded. Let Insurance Companies pay at least fifteen dollars for a surgeon's examination, and insure the surgeon that he shall not be removed for rejecting too many applications, and they will then find thorough examinations, and the rejection of very many applicants that secure the amount of their policies a few months after they have been taken out.

---

A QUEER DISCUSSION.—Several Physicians are quarrelling through the columns of the *Med. and Surg. Reporter*, over the tongues and tails of dogs. One party asserts that there exists a worm, about one inch in length, in the centre of a dog's tongue and in the root of his tail. That these worms are the cause of hydrophobia, and that if they are removed either from tongue or tail, that no dog so operated upon, can have hydrophobia, though bitten by a rabid animal. These physicians, who are scientific gentlemen, worthy of confidence, say that they have operated upon many dogs, have subjected them to the bite of those that were mad, without ever having them show the least sign of hydrophobia.

The opposing party has produced no argument against the theory of the presence of a worm in the tongue and tail of the dog, which gives rise to hydrophobia, but simply ridicules the idea.

We have succeeded in removing the gland containing the perfume of the skunk, but have no desire to search for worms in the tongues of rabid dogs.

FRENCH WINES.—Isn't it a little singular that notwithstanding the immense armies that are marching over and laying waste the celebrated vineyards where most of the fine wines are made, and that little or no wine has been manufactured in France this year, yet the prices on imported (?) wines have not advanced, and the supply is yet more than equal to the demand? Singular, isn't it? This only demonstrates the fact heretofore expressed in the BISTOURY, that most of our supposed imported wines are made in this country, and that we are constantly paying high prices for wines that should and could be furnished us at more moderate rates.

---

COSMETICS.—We published a list of the various nostrums for coloring the hair, in the last BISTOURY, and exhibited the fact that all of them contained lead in varying proportions, which, when absorbed into the circulation through the skin, with which they come in contact, produces paralysis and other disorders. Additional evidence of the baneful character of many of the articles sold as cosmetics, has been developed by the chemical examinations made through the Metropolitan Board of Health of New York. The so-called "enamels" are largely composed of lead, and in one "wash" for the skin was found a large quantity of corrosive sublimate. The supposed innocent white powders for concealing defects in the skin, composed mainly of white clay, will prevent the escape of secretions, and eventually render coarse and injure the surface they were expected to beautify.

---

A HANDSOME AND MERITED REWARD.—One of the most enjoyable gatherings we ever attended, recently occurred in the Hathaway House, for the purpose of presenting a suitable testimonial to the veteran Editor of the *Daily Advertiser*, upon the completion of his twenty-fifth year of editorial labor in this city. We found there assembled, most of the representative men of our city, without regard to politics, who had called to do honor to CHARLES G. FAIRMAN, Esq., the faithful editor, honest gentleman, and exemplary citizen. Hon. S. McDonald made the presentation speech, and handed Mr. Fairman a magnificent, solid silver tea-service, of six pieces, costing over $600.00, which he said, was an offering from his friends of both politial parties, as a slight testimonial of their appreciation of his character as Editor and Citizen.

Mr. Fairman was completely surprised, but responded in a few happy remarks, after which several other citizens were called out through the characteristic remarks of Hon. Luther Caldwell, who, by the way, is always equal to such an emergency. The speeches of Charles Hazard, Esq., of the *Gazette*, and Dr. Pratt of the Corning *Journal* were full of sparkling witticisms, and kept the audiance in good humor until Silas Haight announced a banquet in waiting, which was duly partaken of, and greatly relished, by all present.

We congratulate Mr. Fairman upon the splendid ovation given him, and earnestly hope that he may live long to grace the editorial chair which he has for a quarter of a century, so nobly and worthily filled.

---

MARVELOUS GENIUS.—It is wonderful to witness how perfectly true to nature, even in the smallest details, every incident in the lives of the wonderful characters which DICKENS has created, really are. Particularly is this the case in his description of disease. Every symptom is noted, and as the Physician reads of the last illness of Mrs. Skewton, in "Domby & Son," and his description of epilepsy in Walter Wilding, he is at once astounded at the marvelous medical accuracy with which they are given. One of the finest descriptions of hectic fever may be found in "Oliver Twist," and has really been embodied in more than one standard medical work. It is copied in an English work on Surgery, ("Millers' Principles of Surgery,") and we believe also in Dr. Atkin's Practice of Medicine. This is a great testimony to the wonderful genius of the great novelist.

---

MEDICAL FEES IN PRUSSIA.—A friend, now visiting the Prussian Hospitals and battle fields, sends us the following Fee Bill which is regulated by law, for the guidance of the Physicians of that country, in making their charges for services. After reading it, we doubt whether any Yankee physician could be induced to migrate to that victorious country. For a visit anywhere within a city limits, a physician is allowed to charge one dollar; for each subsequent visit, 25 to 50 cts.; if at a distance of from two to five miles from town and suburbs, his first visit may be from 75 cts. to $1.50, and subsequent ones from 50 to 75 cts. For a first visit at night, if in town, he is allowed $1.50, for more than a mile out of town, from $2.25 to $3.00. He is not allowed to charge for more than two visits a day, to any one patient unless made by special request, and his fees in full for attendance upon any one patient within 24 hours must not exceed $2.25.

